# A New XYZ Compliant Parallel Mechanism for Micro-/Nano-Manipulation: Design and Analysis

**DOI:** 10.3390/mi7020023

**Published:** 2016-02-01

**Authors:** Haiyang Li, Guangbo Hao, Richard C. Kavanagh

**Affiliations:** School of Engineering-Electrical and Electronic Engineering, University College Cork, Cork, Ireland; haiyang.li@umail.ucc.ie (H.L.); r.kavanagh@ucc.ie (R.C.K.)

**Keywords:** compliant parallel mechanism, micro-/nano-manipulation, conceptual design, kinematic analysis, analytical modeling, practical design

## Abstract

Based on the constraint and position identification (CPI) approach for synthesizing XYZ compliant parallel mechanisms (CPMs) and configuration modifications, this paper proposes a new fully-symmetrical XYZ CPM with desired motion characteristics such as reduced cross-axis coupling, minimized lost motion, and relatively small parasitic motion. The good motion characteristics arise from not only its symmetric configuration, but also the rigid linkages between non-adjacent rigid stages. Comprehensive kinematic analysis is carried out based on a series of finite element simulations over a motion range per axis less than ±5% of the beam length, which reveals that the maximum cross-axis coupling rate is less than 0.86%, the maximum lost motion rate is less than 1.20%, the parasitic rotations of the motion stage (MS) are in the order of 10^−5^ rad, and the parasitic translations of the three actuated stages (ASs) are in the order of 10^−4^ of the beam length (less than 0.3% of the motion range), where the beam slenderness ratio is larger than 20. Furthermore, the nonlinear analytical models of the primary translations of the XYZ CPM, including the primary translations of the MS and the ASs, are derived and validated to provide a quick design synthesis. Moreover, two practical design schemes of the proposed XYZ CPM are discussed with consideration of the manufacturability. The practical designs enable the XYZ CPM to be employed in many applications such as micro-/nano-positioning, micro-/nano-manufacturing and micro-/nano-assembly. Finally, a spatial high-precision translational system is presented based on the practical design schemes, taking the actuator and sensor integration into account.

## 1. Introduction

Compliant mechanisms have no traditional sliding or rolling hinges, thereby lending advantages over rigid-body counterparts, such as no backlash, no requirement for lubrication, simplified manufacture, and low part count [1,2,3,4,5,6,7,8]. Currently, XYZ compliant parallel mechanisms (XYZ CPMs) are gaining more and more attention and are being widely employed in a variety of applications such as atomic force microscopy (AFM), micro-/nano-manipulation, high precision alignment, and microelectromechanical systems (MEMS) processes [6,9,10,11,12,13,14,15,16].

In most of the above applications, an XYZ CPM acts as a type of macro-scale mechatronic motion system, and its output motion stage (MS) should be capable of translating along the X-, Y- and Z-axes, with ultra-high precision [17]. However, compliant joints in an XYZ CPM are not ideal compared with their rigid-body counterparts. The stiffness of a compliant joint is neither zero in the degree of freedom (DOF) directions nor infinitely large in the degree of constraint (DOC) directions. Hence, an XYZ CPM often suffers from undesired parasitic motions and coupling motions [9,10]. For the MS of an XYZ CPM as an example, the parasitic motions are the three rotations of the MS about the X-, Y- and Z-axes, which should be much smaller than its three primary translations.

An XYZ CPM is always actuated by three translational actuators, such as voice coils and piezoelectric stacks, exerted on the three actuated stages (ASs: AS-X, AS-Y and AS-Z along the three axes) [5,18]. As is well known, translational actuators cannot tolerate off-axis loads or displacements. Therefore, an AS is permitted to translate only along the actuation direction [9,18], the motions along and about the other directions are the parasitic motions of the AS. In order to protect the three translational actuators and achieve precise control in an XYZ CPM system, the parasitic rotations and the parasitic translations of the three ASs should be minimized.

The three desired translations of the MS and the one permitted translation of each AS in an XYZ CPM system are the primary translations of the XYZ CPM. Based on [9,11], the primary translations of an XYZ CPM should have the following desired characteristics: (a) reduced cross-axis coupling: the primary translation of an MS along one axis should be independent from the primary actuation along the other two axes; and (b) minimized lost motion: in the X-, Y- or Z-axis direction, the motion reduction between the primary translations of the AS and the MS should be minimized. All XYZ CPMs desire the above two primary translation characteristics, especially when closed-loop control systems are not available. The cross-axis coupling of an XYZ CPM can be measured by three cross-axis coupling rates regarding the three primary translations of the MS. When only one axis actuation is applied on an XYZ CPM, the primary translation of the MS along this axis is defined as the nominal primary translation of the MS along the axis. The primary translation of the MS will deviate from the nominal primary translation, if the other one or two actuations are applied. The ratio of the deviation to the nominal primary translation is termed the cross-axis coupling rate along this axis. The lost motions of an XYZ CPM, along the X-, Y- or Z-axes, can be represented as three lost motion rates. The lost motion rate along one axis is defined as the ratio of the motion reduction to the AS’s primary translation along the axis.

As a result, the following attributes, Cross-Axis Coupling Rates, Lost Motion Rates, Parasitic Rotations and Parasitic Translations, can be used to measure the motion performance of an XYZ CPM. This paper defines the motions of an XYZ CPM as follows: MS’s motions along and about the X-, Y- and Z-axes of the global coordinate system are ξ_ms-tx_, ξ_ms-ty_, ξ_ms-tz_, ξ_ms-rx_, ξ_ms-ry_ and ξ_ms-rz_; AS-X’s motions along and about the X-, Y- and Z-axes of the AS-X’s local coordinate system are ξ_asx-tx_, ξ_asx-ty_, ξ_asx-tz_, ξ_asx-rx_, ξ_asx-ry_ and ξ_asx-rz_; AS-Y’s motions along and about the X-, Y- and Z-axes of the AS-Y’s local coordinate system are ξ_asy-tx_, ξ_asy-ty_, ξ_asy-tz_, ξ_asy-rx_, ξ_asy-ry_ and ξ_asy-rz_; AS-Z’s motions along and about the X-, Y- and Z-axes of the AS-Z’s local coordinate system are ξ_asz-tx_, ξ_asz-ty_, ξ_asz-tz_, ξ_asz-rx_, ξ_asz-ry_ and ξ_asz-rz_. Note that the *X*-axes of the AS-X’s, AS-Y’s and AS-Z’s local coordinate systems parallel to the X-, Y- and Z-axes of the global coordinate system, respectively. Therefore, the above four attributes of an XYZ CPM can be represented or calculated, as shown in Table 1. For a desired XYZ CPM, the above four attributes should be minimized. In addition, compact configuration and easy fabrication are also preferable [6].

**Table 1:** Representation or calculation of the four motion characteristics of an XYZ CPM.

Motion Characteristics	X-Axis	Y-Axis	Z-Axis
Cross-Axis Coupling Rates	|(ξ_ms-tx_ − ξ_ms-tx_°)/ξ_ms-tx_°|	|(ξ_ms-ty_ − ξ_ms-ty_°)/ξ_ms-ty_°|	|(ξ_ms-tz_ − ξ_ms-tz_°)/ξ_ms-tz_°|
Lost Motion Rates	(ξ_asx-tx_ − ξ_ms-tx_)/ξ_asx-tx_	(ξ_asy-tx_ − ξ_ms-ty_)/ξ_asy-tx_	(ξ_asz-tx_ − ξ_ms-tx_)/ξ_asz-tx_
Parasitic Rotations	ξ_ms-rx_, ξ_ms-ry_, ξ_ms-rz_, ξ_asx-rx_, ξ_asx-ry_, ξ_asx-rz_, ξ_asy-rx_, ξ_asy-ry_, ξ_asy-rz_, ξ_asz-rx_, ξ_asz-ry_, ξ_asz-rz_		
Parasitic Translations	ξ_asx-ty_, ξ_asx-tz_, ξ_asy-ty_, ξ_asy-tz_, ξ_asz-ty_, ξ_asz-tz_		

Notes: ξ_ms-tx_°, ξ_ms-ty_° and ξ_ms-tz_° represent, respectively, the ξ_ms-tx_ when ξ_ms-ty_ = 0 and ξ_ms-tz_ = 0, the ξ_ms-ty_ when ξ_ms-tx_ = 0 and ξ_ms-tz_ = 0, and the ξ_ms-tz_ when ξ_ms-tx_ = 0 and ξ_ms-ty_ = 0. “| |”denotes the absolute value of a number or a formula between the two vertical bars. For example, |(ξ_ms-tx_ − ξ_ms-tx_°)/ξ_ms-tx_| indicates the absolute value of (ξ_ms-tx_ − ξ_ms-tx_°)/ξ_ms-tx_.

In recent years, many XYZ CPMs [6,9,10,11,12,18,19,20] have been designed using the pseudo-rigid-body-based model substitution approach [21], the constraint-based approach [22,23], the screw-theory-based approach [24], the freedom and constraint topology approach [25,26], and the CPI approach [18]. These existing XYZ CPMs have one or some of the four desired performance characteristics mentioned above. For example, the XYZ CPMs demonstrated in [6,9,10,11,12,18,20] have small cross-axis coupling attributes, and the XYZ CPM reported in [11] also has relative small parasitic rotations. However, none of the reported XYZ CPMs have all the four desired motion characteristics, because the design of such an XYZ CPM is still challenging.

This paper proposes a new XYZ CPM whose cross-axis coupling rates, lost motion rates, parasitic rotations and parasitic translations are minimized. The proposed XYZ CPM is synthesized using the CPI approach [18] with certain configuration modification, such as adding rigid linkages between some non-adjacent rigid stages [27,28]. The good motion characteristics of the designed XYZ CPM are validated by the finite element analysis (FEA) results. Moreover, the primary translations of the XYZ CPM are also nonlinearly modeled in an analytical form, which can offer quick quantitative and parametric insights into the actuation stiffness and the force-displacement relationship.

The remainder of this paper is organized as follows. Section 2 reviews the CPI approach of synthesizing XYZ CPMs. An XYZ CPM, in Section 3, is proposed based on the CPI approach. The kinematic analysis is carried out based on a series of FEA simulations in Section 4. The nonlinear analytical models of the primary translations are obtained and validated in Section 5. Furthermore, Section 6 shows two practical designs of the proposed XYZ CPM, and Section 7 introduces a spatial high-precision translational system designed based on practical designs. Finally, conclusions are drawn in Section 8.

## 2. CPI Approach Revisit

**Figure 1:**
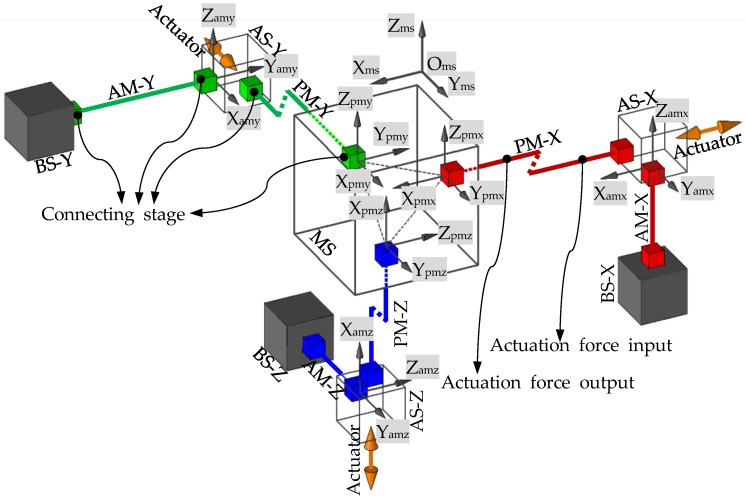
Schematic diagram of a decoupled three-legged XYZ CPM system and the coordinate systems.

The CPI approach proposed in [18] can be employed to synthesize a variety of decoupled XYZ CPMs. The CPI approach decomposes an XYZ CPM into an MS, passive modules (PMs), active modules (AMs), ASs, and base stages (BSs). For a decoupled three-legged XYZ CPM in the CPI approach, the MS, the three PMs (PM-X, PM-Y and PM-Z), the three AMs (AM-X, AM-Y and AM-Z), the three ASs (AS-X, AS-Y and AS-Z), the three BSs (BS-X, BS-Y and BS-Z), and the coordinate systems are defined as demonstrated in Figure 1. The rigid stages, the MS, ASs and BSs, are all assumed to be perfectly rigid, and the compliant modules, AMs and PMs, can be designed on the basis of the constraints selected from the constraint space proposed in the CPI approach. Then, a decoupled three-legged XYZ CPM can be synthesized via assembling all the rigid stages and compliant modules together, based on their position spaces derived via the CPI approach. The constraint space and position space contain many permitted constraints and positions for each compliant module, so that a great number of decoupled three-legged XYZ CPMs can be synthesized using the CPI approach. Additionally, the designed three-legged XYZ CPMs can be further improved, for example by adding redundant legs, so that the resulting XYZ CPMs have good motion characteristics, compact configuration, easy manufacturability, and so on.

## 3. Design of an XYZ CPM

In this section, the design of an XYZ CPM with the four aforementioned motion characteristics is considered. In order to do so, the CPI approach is followed, with consideration of compact configuration and easy manufacturability. The design procedure is demonstrated in the following steps:

1. Select constraints for the PMs and AMs from the constraint space proposed in [18]. Note that the constraints are all described based on the global and local coordinate systems defined in Figure 1. For this case, the selected constraints of the PMs and AMs are illustrated in Table 2. According to the constraints selected, the three PMs or the three AMs in the three legs can be designed with the same structure, because the selected constraints of the PMs or the AMs have the same pattern in their own local coordinate systems.

**Table 2:** Constraints of the passive modules (PMs) and active modules (AMs) in the PM and AM local coordinate systems.

Module	Constraints of the PMs and AMs in the PM and AM Local Coordinate Systems		
	X-Leg	Y-Leg	Z-Leg
PMs	T_pmx-tx_R_pmx-ry_R_pmx-rz_	T_pmy-tx_R_pmy-ry_R_pmy-rz_	T_pmz-tx_R_pmz-ry_R_pmz-rz_
AMs	T_amx-ty_T_amx-tz_R_amx-rx_R_amx-ry_R_ax-rz_	T_amy-ty_T_amy-tz_R_amy-rx_R_amy-ry_R_amy-rz_	T_amz-ty_T_amz-tz_R_amz-rx_R_amz-ry_R_amz-rz_

Notes: T means translational constraint, and R means rotational constraint. The subscribes pmx, pmy and pmz indicate the PM local coordinate systems O_pmx_-X_pmx_Y_pmx_Z_pmx_, O_pmy_-X_pmy_Y_pmy_Z_pmy_ and O_pmz_-X_pmz_Y_pmz_Z_pmz_; and the subscribes amx, amy and amz indicate AM local coordinate systems O_amx_-X_amx_Y_amx_Z_amx_, O_amy_-X_amy_Y_amy_Z_amy_ and O_amz_-X_amz_Y_amz_Z_amz_, respectively. The subscripts -tx, -ty, -tz, -rx, -ry and -rz represent the directions along and about the three orthogonal axes of each coordinate system. For example, T_pmx-tx_ denotes the translational constraint along the X_pmx_-axis of the coordinate system O_pmx_-X_pmx_Y_pmx_Z_pmx_, and R_pmx-ry_ represents the rotational constraint about the Y_pmx_-axis of the coordinate system O_pmx_-X_pmx_Y_pmx_Z_pmx_.

**Figure 2:**
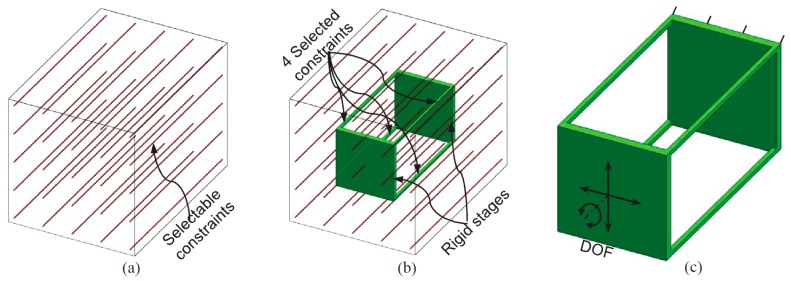
PM design based on the freedom and constraint topology approach: (**a**) the selectable constraints, (**b**) constraint selection and (**c**) the designed PM and its DOF.

**Figure 3:**
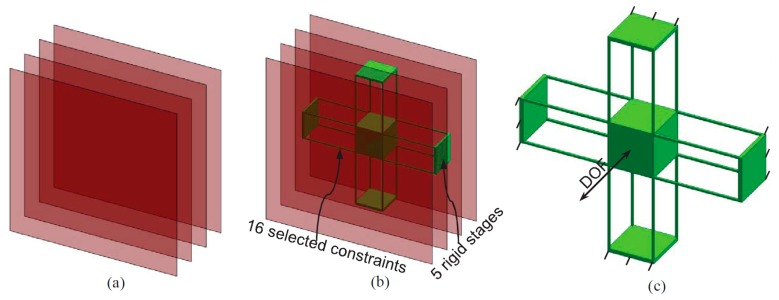
AM design based on the freedom and constraint topology approach: (**a**) the selectable constraints, (**b**) constraint selection and (**c**) the designed AM and its DOF.

**Figure 4:**
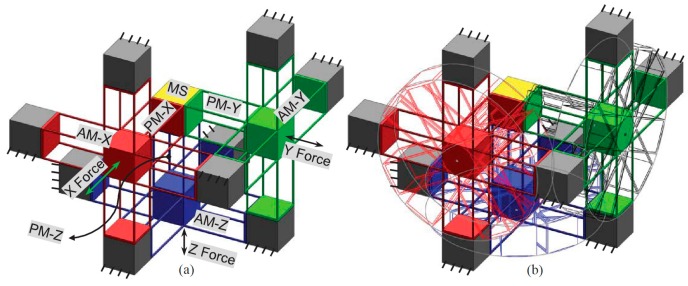
Assembly of the rigid stages and the compliant modules: (**a**) three-legged XYZ CPM assembled, based on the coordinate systems shown in Figure 1 and (**b**) illustration of some permitted positions of the PMs and AMs.

**Figure 5:**
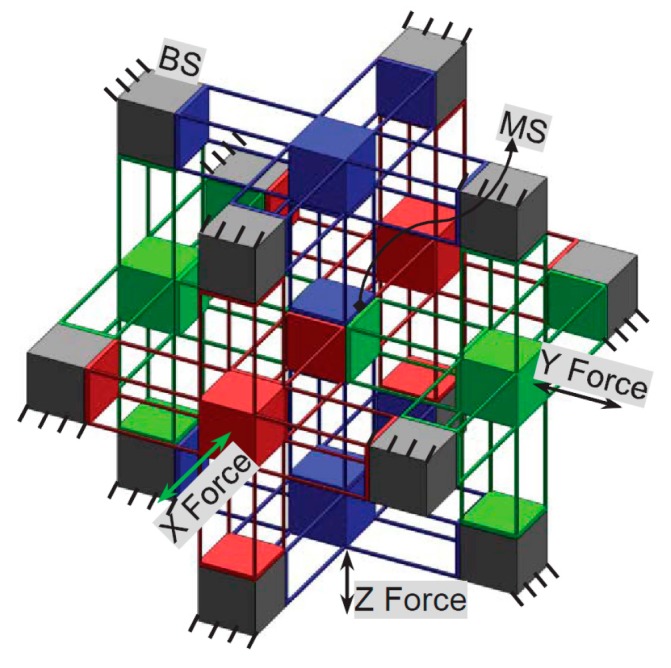
A fully symmetric design, obtained by adding three redundant legs to the XYZ CPM shown in Figure 4a.

**Figure 6:**
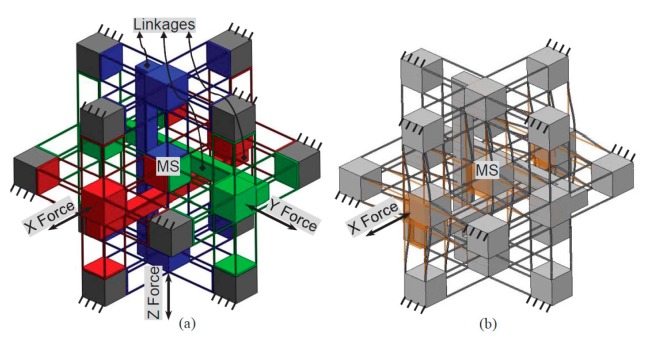
Final design of the XYZ CPM with three rigid linkages: (**a**) the final design and (**b**) the motion along the X-axis.

2. Synthesize the PMs and AMs based on the constraints selected in Step 1 above. In this paper, the PMs and AMs are designed using the freedom and constraint topology approach as shown in Figure 2 and Figure 3. It can be seen that the synthesized PMs and AMs can be fabricated easily.
3. Design the rigid stages, MS, ASs and BSs. Choose cubes as the MS and BSs, and the rigid stages of the AMs can also be used as the three ASs. Note that the exact dimensions of the MS, ASs and BSs can be ignored in this design step.
4. Assemble the PMs, AMs, MS and BSs based on the coordinate systems, as shown in Figure 4a.
5. Identify the final positions for the compliant modules and rigid stages. Using the position space identification method described in [18], the PMs and AMs can translate freely in any direction, and each of the PMs and AMs can also rotate about the X-axis of the local coordinate system, as shown in Figure 4b.The positions of the rigid stages, PMs and AMs, as illustrated in Figure 4a, are chosen as the final positions for the PMs and AMs due to the corresponding easily fabricated structure.
6. Improve the motion performance of the XYZ CPM synthesized in Step 5. The CPI approach can ensure that the designed XYZ CPM in Step 5 has the motion characteristics of kinematic decoupling and actuation isolation. However, the parasitic rotations of the MS are not constrained well. Therefore, three identical redundant legs are added to reduce the parasitic rotations of the MS, as shown in Figure 5.
7. Reduce the lost motion rate between the ASs and the MS. In order to do this, any two ASs associated with the same translation of the MS are rigidly linked, which can be seen in Figure 6a. Two rigidly-linked ASs always have the same motion displacements, because all rigid stages are assumed to be perfectly rigid, as stated in Section 2. Therefore, if one actuation force is exerted on any one of the two linked ASs, the two ASs will move together. Consequently, each translation of the MS is actuated by two ASs in opposite directions simultaneously, which can reduce the lost motion rates between the ASs and the MS. Note that the rigid linkages can also further decouple the primary translations of ASs from each other. In addition, the three rigid linkages can be made of a material with high stiffness and small density, which can reduce the effect of the asymmetric configuration on the XYZ CPM.
8. Check if the XYZ CPM designed in Step 7 meets the design requirements. The synthesized XYZ CPM, as shown in Figure 6, has the desired characteristics, so it is the final design.

## 4. FEA-Result-Based Kinematic Analysis

In this section, the motion characteristics, including the cross-axis coupling rates, the lost motion rates, the parasitic rotations and the parasitic translations of the XYZ CPM are analyzed based on the FEA results. And the relations between the motion characteristics and the geometric parameters are studied. In addition, the comparison between the two XYZ CPMs with and without the rigid linkages, as shown in Figure 5 and Figure 6, is also carried out.

The designed XYZ CPM, as shown in Figure 6, has an isotropic configuration (all the wire beams of the XYZ CPM are identical in dimension and material), so only the motion characteristics associated with one of the three orthogonal directions needs to be analyzed. In this paper, the motion characteristics associated with the X-axis translation is studied. The rotational stiffness of the ASs is larger than those of the MS, due to introducing the three rigid linkages. So the parasitic rotations of the ASs should not be larger than the parasitic rotations of the MS. Therefore, the parasitic rotations of the ASs are not analyzed in this case. As a result, only the primary translations of the MS and the AS-X, the parasitic rotations of the MS, and the parasitic translations of the AS-X are studied in this case. Additionally, in this paper, the displacements and lengths are normalized by the beam length *L*, the translational forces are normalized by *EI*/*L*^2^, and the rotational forces are normalized by *EI*/*L*, where *E* is the Young’s modulus of the material, and *I* is the second moment of area of the beam [29].

For the FEA model, assume that *E =* 69,000 MPa, *υ* = 0.33, and the normalized motion range per axis is limited to ±5%*L* which can be considered as a medium-large motion range compared with the beam length [29]. Moreover, the commercial software, COMSOL MULTIPHYSICS (COMSOL Group, Stockholm, Sweden), is selected for the nonlinear FEA simulations, using the 10-node tetrahedral element and fine meshing technology. In addition, the three actuations are applied on the surface centers of the three cubic ASs, respectively, as shown in Figure 6. Additionally, each of the three actuation points is the motion reference point of the AS in the FEA simulations, whose motion is regarded as the motion of the AS. Similarly, the center point of the cubic MS is defined as the motion reference point of the MS.

The main geometric parameters of the XYZ CPM are the beam length *L*, the beam thickness *T* and the width *W* of the identical cubes, which can be analyzed in the following two dimensionless parameters: beam’s slenderness ratio λ_b_ (in this paper, λ_b_* = L*/*T*) and the ratio, *w*, of the *W* to the *L* (*w* is actually the normalized width of the identical cubes*,* which equals to *W*/*L*). In order to estimate the effects of the two dimensionless parameters on the motion characteristics, a series of values are selected for the two dimensionless parameters, λ_b_ and *w*, in the FEA model.

In order to achieve a several-millimeter motion range, the beam length *L* should be up to dozens of millimeters (as mentioned above, the motion range per axis equals to ±5%*L*). So it is appropriate to select 20, 30, 40, 50, 60, 70, 80, 90 and 100 mm as the beam length, respectively. For these beam lengths, 1 mm beam thickness and 20 mm width are appropriate. Based on the above selected dimension, λ_b_ varies from 20 to 100. For each of the values of λ_b_, FEA simulation is carried out, under the following actuation condition: The normalized X-axis actuation displacement, ξ_asx-tx_, varies from −0.05 to +0.05, and the other two normalized actuation displacements, ξ_asy-tx_ and ξ_asz-tx_, are both 0.05. In this actuation condition, the cross-axis coupling rate regarding the MS’s X-axis translation, the lost motion rate along the X-axis, the parasitic rotations of the MS about the Y- and Z-axes, and the parasitic translations of the AS-X along the Y- and Z-axes can reach their maximum values.

**Figure 7:**
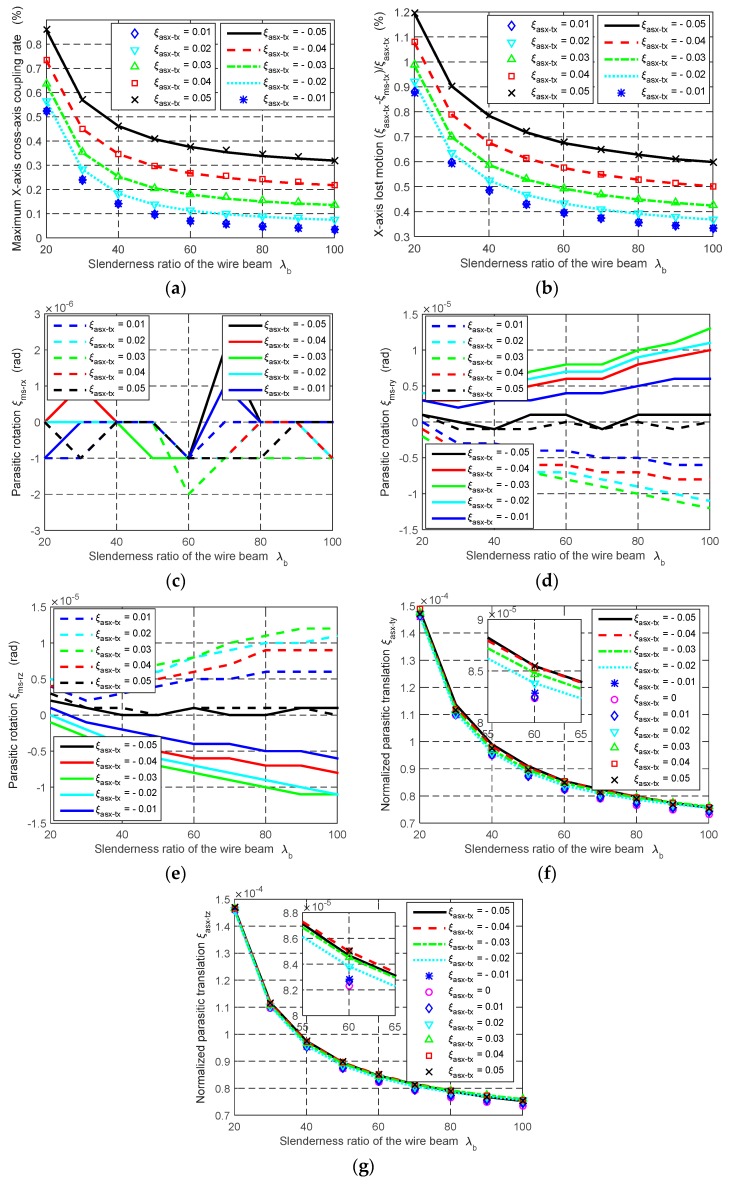
The variation of the motion characteristics of the XYZ CPM with the beam slenderness ratios λ_b_: (**a**) Cross-axis coupling rate in terms of the X-axis primary translation of the MS, (**b**) lost motion rate along the X-axis, (**c**–**e**) parasitic rotations of the MS about the X-, Y- and Z-axes, respectively and (**f**–**g**) parasitic translations of the AS along the Y- and Z-axes, respectively.

The FEA results are shown in Figure 7. With λ_b_ changing from 20 to 100, it can be seen that: (a) the maximum cross-axis coupling rate, in terms of the MS’s X-axis translation, reduces from 0.86% to 0.32%, as shown in Figure 7a; (b) the maximum X-axis lost motion rate varies from 1.20% to 0.60%, as shown in Figure 7b; (c) the parasitic rotation of the MS about the X-axis shows a highly discontinuous behavior responding to the slenderness ratio conditions, which are in the order of 10^−6^ rad, as shown in Figure 7c; (d) the maximum parasitic rotation of the MS about the Y- or Z-axis increases slightly, but is still in the order of 10^−5^, as shown in Figure 7d,e; and (e) the normalized parasitic translations of the AS-X along the Y- and Z-axes decrease from about 1.5 × 10^−4^ to 0.76 × 10^−4^, as shown in Figure 7f,g. In the condition of increasing the value of λ_b_, the FEA results reveal that the cross-axis coupling rate, the lost motion rate and the parasitic translations decrease, but the parasitic rotations increase slightly. It can also be seen that the obtained cross-axis coupling rate, lost motion rate, parasitic rotations and parasitic translations are reduced compared with the XYZ CPM proposed in [9], for all the values of λ_b_.

Compared with the effect of λ_b_ under the same actuation condition, the effect of *w* on the motion characteristics of the XYZ CPM is negligible, which can be seen from the FEA results shown in Table 3 (when *L* = 30 mm). Particularly, the cross-axis coupling rate and the lost motion rate do not change, when the *w* are assigned 1/3, 2/3 and 1, respectively. The parasitic rotations decrease slightly when increasing the *w* value.

**Figure 8:**
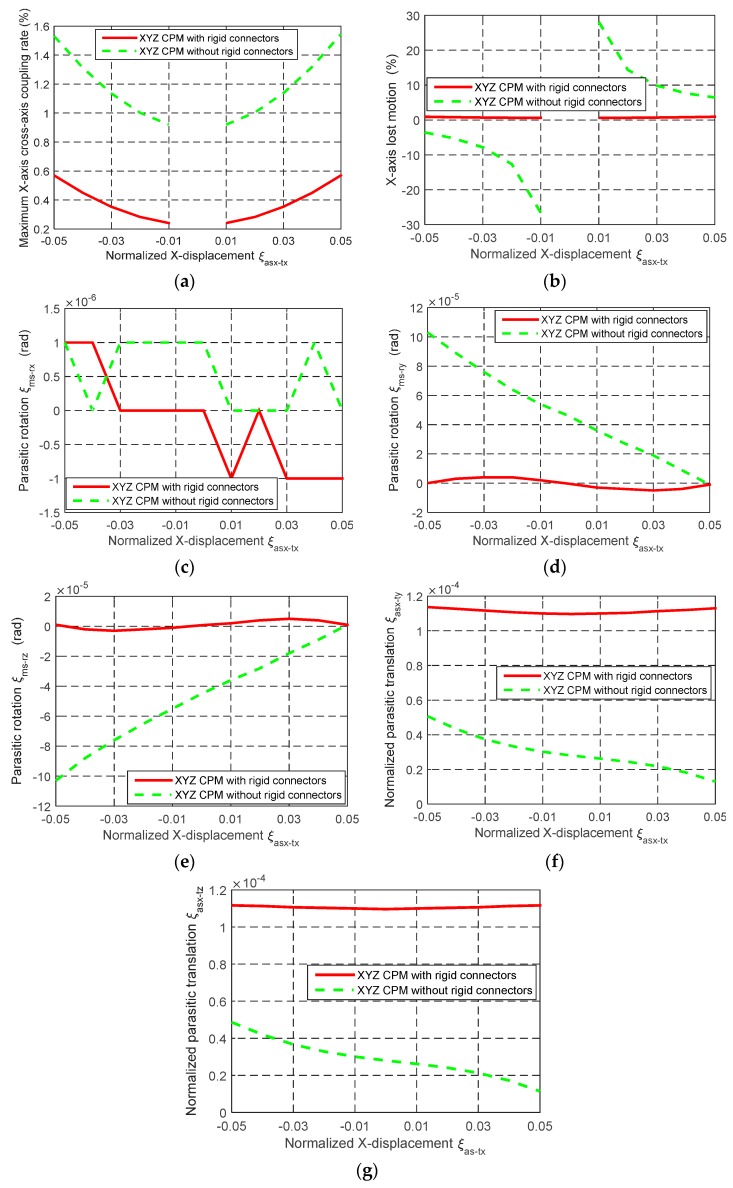
The comparison of the motion characteristics of the two XYZ CPMs with and without the rigid linkages, as shown in Figure 5 and Figure 6, respectively: (**a**) Cross-axis coupling rate in terms of the X-axis primary translation of the MS, (**b**) lost motion rate along the X-axis, (**c**–**e**) parasitic rotations of the MS about the X-, Y- and Z-axes; respectively and (**f**–**g**) parasitic translations of the AS along the Y- and Z-axes, respectively.

The comparison of the motion characteristics of the two XYZ CPMs with and without the rigid linkages, as shown in Figure 5 and Figure 6, is also conducted in this section. When *λ*_b_ = 30 and *w* = 2/3, the comparison of the motion characteristics of the two XYZ CPMs is demonstrated in Figure 8. The FEA results illustrate that: (a) the cross-axis coupling rate, the lost motion rate and the parasitic rotations of the XYZ CPM with the rigid linkages are much smaller than corresponding ones of the XYZ CPM without the rigid linkages, respectively; and (b) the AS-X of the XYZ CPM with the rigid linkages has slightly larger normalized parasitic translations, which are, however, in the order of 10^−4^ and less than 0.23% of the primary translation of the AS-X. For any one of the two XYZ CPMs with and without the rigid linkages, the parasitic translations of the AS-X along the Y- and Z-axes mainly arise from the actuation forces along the Y- and Z-axes, respectively. The parasitic translations of the AS-X of the XYZ CPM without the rigid linkages along the Y- and Z-axes are slightly smaller than those of the XYZ CPM with the rigid linkages, probably because, for a certain displacement of the AS-X, the XYZ CPM without the rigid linkages needs a smaller actuation force along the X-axis.

**Table 3:** The variation of the motion characteristics of the XYZ CPM with the rigid-cube width *w.*

AS-X X-Translation, ξ_asx-tx_		−0.05	−0.03	−0.01	0.01	0.03	0.05
Cross-axis coupling rate (%)	*w* = 1/3	0.57	0.35	0.24	0.24	0.35	0.57
	*w* = 2/3	0.57	0.35	0.24	0.24	0.35	0.57
	*w* = 1	0.57	0.35	0.24	0.24	0.35	0.57
Lost motion rate (%)	*w* = 1/3	0.90	0.70	0.59	0.59	0.70	0.90
	*w* = 2/3	0.90	0.70	0.59	0.60	0.70	0.90
	*w* = 1	0.90	0.70	0.59	0.60	0.70	0.90
MS X-rotation, ξ_ms-rx_, (rad)	*w* = 1/3	0.00 × 10^0^	0.00 × 10^0^	−1.00 × 10^−6^	−1.00 × 10^−6^	0.00 × 10^0^	−1.00 × 10^−6^
	*w* = 2/3	1.00 × 10^−6^	0.00 × 10^0^	0.00 × 10^0^	−1.00 × 10^−6^	−1.00 × 10^−6^	−1.00 × 10^−6^
	*w* = 1	−3.00 × 10^−6^	−2.00 × 10^−6^	−1.00 × 10^−6^	−1.00 × 10^−6^	−1.00 × 10^−6^	−1.00 × 10^−6^
MS Y-rotation, ξ_ms-ry_, (rad)	*w* = 1/3	0.00 × 10^0^	6.00 × 10^−6^	3.00 × 10^−6^	−3.00 × 10^−6^	−6.00 × 10^−6^	0.00 × 10^0^
	*w* = 2/3	0.00 × 10^0^	4.00 × 10^−6^	2.00 × 10^−6^	−3.00 × 10^−6^	−5.00 × 10^−6^	−1.00 × 10^−6^
	*w* = 1	0.00 × 10^0^	4.00 × 10^−6^	2.00 × 10^−6^	−1.00 × 10^−6^	−2.00 × 10^−6^	2.00 × 10^−6^
MS Z-rotation, ξ_ms-rz_, (rad)	*w* = 1/3	0.00 × 10^0^	−5.00 × 10^−6^	−3.00 × 10^−6^	3.00 × 10^−6^	5.00 × 10^−6^	0.00 × 10^0^
	*w* = 2/3	1.00 × 10^−6^	−3.00 × 10^−6^	−1.00 × 10^−6^	2.00 × 10^−6^	5.00 × 10^−6^	1.00 × 10^−6^
	*w* = 1	1.00 × 10^−6^	−4.00 × 10^−6^	−2.00 × 10^−6^	1.00 × 10^−6^	3.00 × 10^−6^	0.00 × 10°
AS-X Y-translation, ξ_asx-ty_	*w* = 1/3	1.12 × 10^−4^	1.11 × 10^−4^	1.09 × 10^−4^	1.09 × 10^−4^	1.10 × 10^−4^	1.11 × 10^−4^
	*w* = 2/3	1.14 × 10^−4^	1.12 × 10^−4^	1.10 × 10^−4^	1.10 × 10^−4^	1.11 × 10^−4^	1.13 × 10^−4^
	*w* = 1	1.11 × 10^−4^	1.10 × 10^−4^	1.09 × 10^−4^	1.09 × 10^−4^	1.10 × 10^−4^	1.11 × 10^−4^
AS-X Z-translation, ξ_asx-tz_	*w* = 1/3	1.12 × 10^−4^	1.11 × 10^−4^	1.10 × 10^−4^	1.10 × 10^−4^	1.11 × 10^−4^	1.12 × 10^−4^
	*w* = 2/3	1.12 × 10^−4^	1.11 × 10^−4^	1.10 × 10^−4^	1.10 × 10^−4^	1.11 × 10^−4^	1.12 × 10^−4^
	*w* = 1	1.11 × 10^−4^	1.10 × 10^−4^	1.09 × 10^−4^	1.09 × 10^−4^	1.10 × 10^−4^	1.11 × 10^−4^

## 5. Analytical Modeling

The motion characteristics of the XYZ CPM, as shown in Figure 6, are predicted based on the FEA results, and the relations between the motion characteristics and the main geometric parameters are studied as well. In this section, the nonlinear analytical model of the XYZ CPM is derived, which can be used to predict the force-displacement relationship, and to estimate the actuation stiffness, before conducting FEA simulations and experimental tests. As observed in Section 4, the parasitic rotations and the parasitic translations are much smaller than the primary translations. Therefore, it is appropriate to ignore the effects of the parasitic motion on the analytical models of the primary translations.

**Figure 9:**
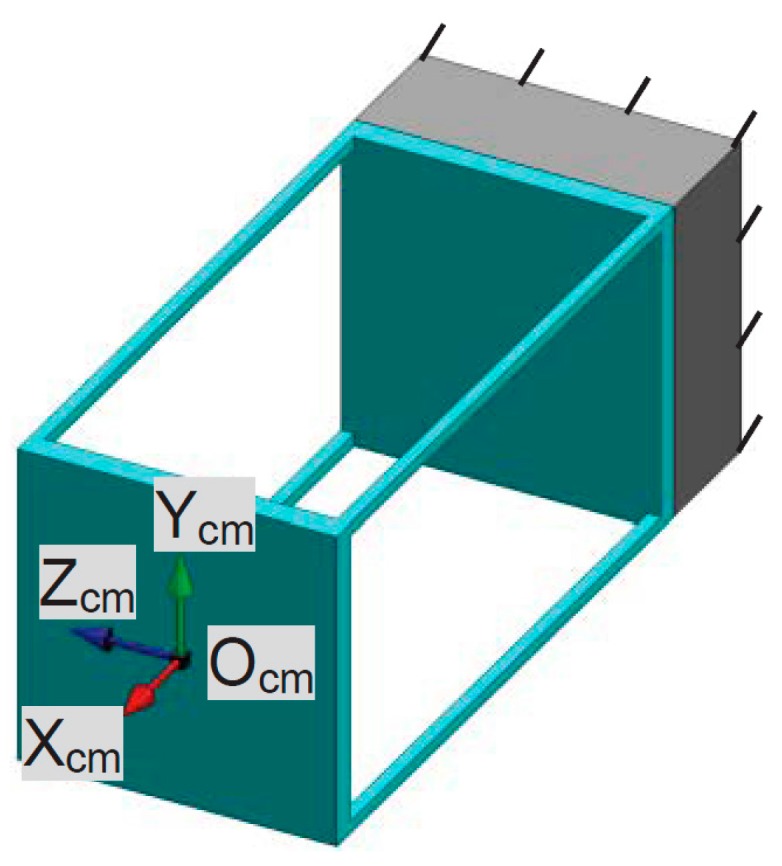
An FBCM and its coordinate system.

Each PM of the XYZ CPM can be referred to as a four-beam compliant module (FBCM) as shown in Figure 9, and each AM of the XYZ CPM can be regarded as the parallel combination of four FBCMs. Therefore, the motions of the XYZ CPM are performed through the deformation of the FBCMs in the XYZ CPM, so the modeling of the FBCM is carried out before modeling the complete XYZ CPM.

The analytical model of the FBCM can be found in [30,31], which can be rewritten as Equations (1)–(3).

(1) $$\zeta_{\mathrm{cm}-\mathrm{tx}}=-\frac{1680\left(5\xi_{\mathrm{cm}-\mathrm{tx}}+3\left(\xi_{\mathrm{cm}-\mathrm{ty}}^{2}+\xi_{\mathrm{cm}-\mathrm{tz}}^{2}\right)\right)}{175t^{2}+3\xi_{\mathrm{cm}-\mathrm{ty}}^{2}+3\xi_{\mathrm{cm}-\mathrm{tz}}^{2}}$$

(2) $$\zeta_{\text{cm}-\text{ty}}=-\frac{48\xi_{\mathrm{cm}-\mathrm{ty}}\left(175t^{2}+210\xi_{\mathrm{cm}-\mathrm{tx}}+129\xi_{\mathrm{cm}-\mathrm{ty}}^{2}+129\xi_{\mathrm{cm}-\mathrm{tz}}^{2}\right)}{175t^{2}+3\xi_{\mathrm{cm}-\mathrm{ty}}^{2}+3\xi_{\mathrm{cm}-\mathrm{tz}}^{2}}$$

(3) $$\zeta_{\text{cm}-\text{tz}}=-\frac{48\xi_{\mathrm{cm}-\mathrm{tz}}\left(175t^{2}+210\xi_{\mathrm{cm}-\mathrm{tx}}+129\xi_{\mathrm{cm}-\mathrm{ty}}^{2}+129\xi_{\mathrm{cm}-\mathrm{tz}}^{2}\right)}{175t^{2}+3\xi_{\mathrm{cm}-\mathrm{ty}}^{2}+3\xi_{\mathrm{cm}-\mathrm{tz}}^{2}}$$

where *t* is normalized thickness of the beam. ξ_cm-tx_, ξ_cm-ty_ and ξ_cm-tz_ are the translational displacements of the FBCM’s output stage along the X_cm_-, Y_cm_- and Z_cm_-axes, respectively. ζ_cm-tx_, ζ_cm-ty_ and ζ_cm-tz_ are the reaction forces along the X_cm_-, Y_cm_- and Z_cm_-axes, respectively, which are produced by the FBCM due to the deformation of the beams in the FBCM. Note that the rotations of the FBCM about the X_cm_-, Y_cm_- and Z_cm_-axes are not taken into account, because the rotations of the FBCM in the XYZ CPM system are negligible compared with the translations, as analyzed in Section 4.

**Figure 10:**
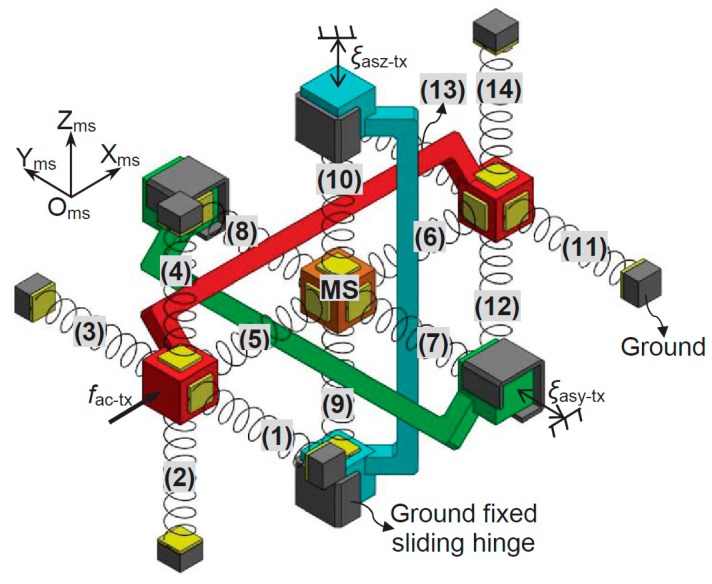
Demonstration of the rigid stages and FBCMs in the XYZ CPM system associated with only the X_ms_-axis translation (AS-Xs and their rigid linkage are red in color, AS-Ys and their rigid linkage are green in color, and AS-Zs and their rigid linkage are blue in color).

According to the analytical model, an FBCM can be regarded as a three-dimension translational spring. The complete XYZ CPM can be modeled based on the analytical model of the three-dimension translational spring. As already mentioned, the motion performance of the XYZ CPM along the X_ms_-, Y_ms_- and Z_ms_-axes are identical (O_ms_-X_ms_Y_ms_Z_ms_ are the global coordinate system in this section). Therefore, only the primary translations along one of the three directions need to be studied. In this paper, the derivation of the force-displacement relationship, associated with only the translations along the X_ms_-axis, is detailed. Given any displacements, ξ_asy-tx_ and ξ_asz-tx_, of the AS-Y and AS-Z, respectively, the XYZ CPM can be simplified to the model shown in Figure 10 if only the force-displacement relationship in X_m_-axis is concerned.

Assume that the lost motions along the X_ms_-, Y_ms_- and Z_ms_-axes are δ_x_, δ_y_ and δ_z_, respectively, which can be written as shown in Equation (4).

(4) $$\delta_{x}=\xi_{\mathrm{asx}-\mathrm{tx}}-\xi_{\mathrm{ms}-\mathrm{tx}},\;\delta_{y}=\xi_{\mathrm{asy}-\mathrm{tx}}-\xi_{\mathrm{ms}-\mathrm{ty}}\;\mathrm{and}\;\delta_{z}=\xi_{\mathrm{asz}-\mathrm{tx}}-\xi_{\mathrm{ms}-\mathrm{tz}}$$

where ξ_ms-tx_, ξ_ms-ty_, ξ_ms-tz_, ξ_asx-tx_, ξ_asy-tx_ and ξ_asz-tx_ are the primary translations of the MS and ASs, as defined in Section 1. The model, as shown in Figure 10, contains 14 FBCMs, which are termed as FBCM-1 to FBCM-14, respectively. If all the parasitic rotations and parasitic translations of the XYZ CPM are ignored, the deformation displacements of each of the FBCMs can be obtained easily according to the primary translations and lost motions. Additionally, the reaction forces of the FBCMs can also be calculated based on Equation (1). Taking the FBCM-1 as an example, the FBCM-1 is linked to the AS-X, so the deformation displacements of the FBCM-1 can be derived from the motion displacements of the AS-X. If ignoring all the parasitic rotations and parasitic translations of the AS-X, the deformation displacements of the FBCM-1 equal to ξ_asx-tx_, zero and zero along the X_ms_-, Y_ms_- and Z_ms_-axes, respectively. Therefore, the reaction force, ζ_a_, of the FBCM-1 along the X_ms_-axis can be obtained, as shown in Equation (5), via substituting the deformation displacements of the FBCM-1 into Equations (2) or (3). Note that when substituting the deformation displacements into Equation (2), ξ_cm-tx_, ξ_cm-ty_ and ξ_cm-tz_ in Equation (2) equal to zero, ξ_asx-tx_ and zero, respectively; when substituting the deformation displacements into Equation (3), ξ_cm-tx_, ξ_cm-ty_ and ξ_cm-tz_ in Equation (3) equal to zero, zero and ξ_asx-tx_, respectively. Similarly, the reaction force of the FBCM-2, FBCM-3, FBCM-4, FBCM-11, FBCM-12, FBCM-13 or FBCM-14, to the AS-X along the X_ms_-axis, can also be obtained as shown in Equation (5). Additionally, the reaction forces of the FBCM-5, FBCM-6, FBCM-7, FBCM-8, FBCM-9 and FBCM-10, to the MS along the X_ms_-axis, can be derived in Equations (6)–(11), respectively, which are represented as ζ_b_, ζ_c_, ζ_d_, ζ_e_, ζ_f_ and ζ_g_.

(5) $$\zeta_{a}=-\frac{48\xi_{\mathrm{asx}-\mathrm{tx}}\left(129\xi_{\mathrm{asx}-\mathrm{tx}}^{2}+175t^{2}\right)}{3\xi_{\mathrm{asx}-\mathrm{tx}}^{2}+175t^{2}}$$

(6) $$\zeta_{b}=\frac{1680\left(3\left(\xi_{\text{ms}-\text{ty}}^{2}+\xi_{\text{ms}-\text{tz}}^{2}\right)-5\delta_{x}\right)}{3\xi_{\text{ms}-\text{ty}}^{2}+3\xi_{\text{ms}-\text{tz}}^{2}+175t^{2}}$$

(7) $$\zeta_{c}=-\frac{1680\left(3\left(\xi_{\mathrm{ms}-\mathrm{ty}}^{2}{\text{+ξ}}_{\mathrm{ms}-\mathrm{tz}}^{2}\right)-5\delta_{x}\right)}{3\xi_{\mathrm{ms}-\mathrm{ty}}^{2}+3\xi_{\mathrm{ms}-\mathrm{tz}}^{2}+175t^{2}}$$

(8) $$\zeta_{d}=-\frac{48\xi_{\mathrm{ms}-\mathrm{tx}}\left(-210\delta_{y}+129\xi_{\mathrm{ms}-\mathrm{tx}}^{2}+129\xi_{\mathrm{ms}-\mathrm{tz}}^{2}+175t^{2}\right)}{3\xi_{\mathrm{ms}-\mathrm{tx}}^{2}+3\xi_{\mathrm{ms}-\mathrm{tz}}^{2}+175t^{2}}$$

(9) $$\zeta_{e}=-\frac{48\xi_{\mathrm{ms}-\mathrm{tx}}\left(210\delta_{y}+129\xi_{\mathrm{ms}-\mathrm{tx}}^{2}+129\xi_{\mathrm{ms}-\mathrm{tz}}^{2}+175t^{2}\right)}{3\xi_{\mathrm{ms}-\mathrm{tx}}^{2}+3\xi_{\mathrm{ms}-\mathrm{tz}}^{2}+175t^{2}}$$

(10) $$\zeta_{f}=-\frac{48\xi_{\mathrm{ms}-\mathrm{tx}}\left(-210\delta_{z}+129\xi_{\mathrm{ms}-\mathrm{ty}}^{2}+129\xi_{\mathrm{ms}-\mathrm{tx}}^{2}+175t^{2}\right)}{3\xi_{\mathrm{ms}-\mathrm{ty}}^{2}+3\xi_{\mathrm{ms}-\mathrm{tx}}^{2}+175t^{2}}$$

(11) $$\zeta_{g}=-\frac{48\xi_{\mathrm{ms}-\mathrm{tx}}\left(210\delta_{z}+129\xi_{\mathrm{ms}-\mathrm{ty}}^{2}+129\xi_{\mathrm{ms}-\mathrm{tx}}^{2}+175t^{2}\right)}{3\xi_{\mathrm{ms}-\mathrm{ty}}^{2}+3\xi_{\mathrm{ms}-\mathrm{tx}}^{2}+175t^{2}}$$

When the MS is at static equilibrium, all the reaction forces on the MS along the X_ms_-axis should be balanced, so Equation (12) can be obtained. Substituting Equations (6)–(11) into Equation (12), Equation (13) can be derived. Furthermore, the actuation force, *f*_ac-tx_, should equal to the sum of the reaction forces of all the FBCMs except FBCM-5 and FBCM-6, along the X_ms_-axis. Therefore, the relationship between the actuation force *f*_ac-tx_ and the primary translations of the MS can be obtained, as shown in Equation (14).

(12) $$\zeta_{b}+\zeta_{c}+\zeta_{d}+\zeta_{e}+\zeta_{f}+\zeta_{g}=0$$

(13) $$\delta_{x}=\frac{2\xi_{\mathrm{ms}-\mathrm{tx}}(3\xi_{\mathrm{ms}-\mathrm{ty}}^{2}+3\xi_{\mathrm{ms}-\mathrm{tz}}^{2}+175t^{2})\left(3\xi_{\mathrm{ms}-\mathrm{tx}}^{2}\left(129\xi_{\mathrm{ms}-\mathrm{ty}}^{2}+129\xi_{\mathrm{ms}-\mathrm{tz}}^{2}+7700t^{2}\right)+175\left({\text{66t}}^{2}\xi_{\mathrm{ms}-\mathrm{tz}}^{2}+175t^{4}\right)\right)}{175\left(3\xi_{\mathrm{ms}-\mathrm{tx}}^{2}+3\xi_{\mathrm{ms}-\mathrm{ty}}^{2}+175t^{2}\right)\left(3\xi_{\mathrm{ms}-\mathrm{tx}}^{2}+3\xi_{\mathrm{ms}-\mathrm{tz}}^{2}+175t^{2}\right)}+\frac{6\xi_{\mathrm{ms}-\mathrm{tx}}\left(3\xi_{\mathrm{ms}-\mathrm{ty}}^{2}+3\xi_{\mathrm{ms}-\mathrm{tz}}^{2}+175t^{2}\right)\left(\xi_{\mathrm{ms}-\mathrm{ty}}^{2}\left(129\xi_{\mathrm{ms}-\mathrm{tz}}^{2}+3850t^{2}\right)+129\xi_{\mathrm{ms}-\mathrm{tx}}^{4}\right)}{175\left(3\xi_{\mathrm{ms}-\mathrm{tx}}^{2}+3\xi_{\mathrm{ms}-\mathrm{ty}}^{2}+175t^{2}\right)\left(3\xi_{\mathrm{ms}-\mathrm{tx}}^{2}+3\xi_{\mathrm{ms}-\mathrm{tz}}^{2}+175t^{2}\right)}$$

(14) $$f_{\mathrm{ac}-\mathrm{tx}}=96\xi_{\mathrm{ms}-\mathrm{tx}}\left(\frac{516\xi_{\mathrm{ms}-\mathrm{tx}}^{2}+700t^{2}}{3\xi_{\mathrm{ms}-\mathrm{tx}}^{2}+175t^{2}}+\frac{129\xi_{\mathrm{ms}-\mathrm{tx}}^{2}+129\xi_{\mathrm{ms}-\mathrm{ty}}^{2}+175t^{2}}{3\xi_{\mathrm{ms}-\mathrm{tx}}^{2}+3\xi_{\mathrm{ms}-\mathrm{ty}}^{2}+175t^{2}}+\frac{129\xi_{\mathrm{ms}-\mathrm{tx}}^{2}+129\xi_{\mathrm{ms}-\mathrm{tz}}^{2}+175t^{2}}{3\xi_{\mathrm{ms}-\mathrm{tx}}^{2}+3\xi_{\mathrm{ms}-\mathrm{tz}}^{2}+175t^{2}}\right)$$

Similarly, the lost motions along the Y_ms_- and Z_ms_-axes and the force-displacement relationships associated with the actuation forces, *f*_ac-ty_ and *f*_ac-tz_, can be derived as shown in Equations (15)–(18). Note that the actuation forces, *f*_ac-ty_ and *f*_ac-tz_, are applied on the AS-Y and AS-Z, respectively.

(15) $$\begin{array}{c} \delta_{y}=\frac{2\xi_{\mathrm{ms}-\mathrm{ty}}\left(3\xi_{\mathrm{ms}-\mathrm{tx}}^{2}+3\xi_{\mathrm{ms}-\mathrm{tz}}^{2}+175t^{2}\right)\left(3\xi_{\mathrm{ms}-\mathrm{ty}}^{2}\left(129\xi_{\mathrm{ms}-\mathrm{tx}}^{2}+129\xi_{\mathrm{ms}-\mathrm{tz}}^{2}+7700t^{2}\right)+175\left({\text{66t}}^{2}\xi_{\mathrm{ms}-\mathrm{tz}}^{2}+175t^{4}\right)\right)}{175\left(3\xi_{\mathrm{ms}-\mathrm{tx}}^{2}+3\xi_{\mathrm{ms}-\mathrm{ty}}^{2}+175t^{2}\right)\left(3\xi_{\mathrm{ms}-\mathrm{ty}}^{2}+3\xi_{\mathrm{ms}-\mathrm{tz}}^{2}+175t^{2}\right)}+\frac{6\xi_{\mathrm{ms}-\mathrm{ty}}\left(3\xi_{\mathrm{ms}-\mathrm{tx}}^{2}+3\xi_{\mathrm{ms}-\mathrm{tz}}^{2}+175t^{2}\right)\left(\xi_{\mathrm{ms}-\mathrm{tx}}^{2}\left(129\xi_{\mathrm{ms}-\mathrm{tz}}^{2}+3850t^{2}\right)+129\xi_{\mathrm{ms}-\mathrm{ty}}^{4}\right)}{175\left(3\xi_{\mathrm{ms}-\mathrm{tx}}^{2}+3\xi_{\mathrm{ms}-\mathrm{ty}}^{2}+175t^{2}\right)\left(3\xi_{\mathrm{ms}-\mathrm{ty}}^{2}+3\xi_{\mathrm{ms}-\mathrm{tz}}^{2}+175t^{2}\right)} \end{array}$$

(16) $$f_{\mathrm{ac}-\mathrm{ty}}={\text{96ξ}}_{\mathrm{ms}-\mathrm{ty}}\left(\frac{516\xi_{\mathrm{ms}-\mathrm{ty}}^{2}+700t^{2}}{3\xi_{\mathrm{ms}-\mathrm{ty}}^{2}+175t^{2}}+\frac{129\xi_{\mathrm{ms}-\mathrm{tx}}^{2}+129\xi_{\mathrm{ms}-\mathrm{ty}}^{2}+175t^{2}}{3\xi_{\mathrm{ms}-\mathrm{tx}}^{2}+3\xi_{\mathrm{ms}-\mathrm{ty}}^{2}+175t^{2}}+\frac{129\xi_{\mathrm{ms}-\mathrm{ty}}^{2}+129\xi_{\mathrm{ms}-\mathrm{tz}}^{2}+175t^{2}}{3\xi_{\mathrm{ms}-\mathrm{ty}}^{2}+3\xi_{\mathrm{ms}-\mathrm{tz}}^{2}+175t^{2}}\right)$$

(17) $$\begin{array}{c} \delta_{z}=\frac{2\xi_{\mathrm{ms}-\mathrm{tz}}\left(3\xi_{\mathrm{ms}-\mathrm{tx}}^{2}+3\xi_{\mathrm{ms}-\mathrm{ty}}^{2}+175t^{2}\right)\left(3\xi_{\mathrm{ms}-\mathrm{tz}}^{2}\left(129\xi_{\mathrm{ms}-\mathrm{tx}}^{2}+129\xi_{\mathrm{ms}-\mathrm{ty}}^{2}+7700t^{2}\right)+175\left({\text{66t}}^{2}\xi_{\mathrm{ms}-\mathrm{tx}}^{2}+175t^{4}\right)\right)}{175\left(3\xi_{\mathrm{ms}-\mathrm{ty}}^{2}+3\xi_{\mathrm{ms}-\mathrm{tz}}^{2}+175t^{2}\right)\left(3\xi_{\mathrm{ms}-\mathrm{tx}}^{2}+3\xi_{\mathrm{ms}-\mathrm{tz}}^{2}+175t^{2}\right)}+\frac{6\xi_{\mathrm{ms}-\mathrm{tz}}\left(3\xi_{\mathrm{ms}-\mathrm{tx}}^{2}+3\xi_{\mathrm{ms}-\mathrm{ty}}^{2}+175t^{2}\right)\left(\xi_{\mathrm{ms}-\mathrm{ty}}^{2}\left(129\xi_{\mathrm{ms}-\mathrm{tx}}^{2}+3850t^{2}\right)+129\xi_{\mathrm{ms}-\mathrm{tz}}^{4}\right)}{175\left(3\xi_{\mathrm{ms}-\mathrm{ty}}^{2}+3\xi_{\mathrm{ms}-\mathrm{tz}}^{2}+175t^{2}\right)\left(3\xi_{\mathrm{ms}-\mathrm{tx}}^{2}+3\xi_{\mathrm{ms}-\mathrm{tz}}^{2}+175t^{2}\right)} \end{array}$$

(18) $$f_{\mathrm{ac}-\mathrm{tz}}=96\xi_{\mathrm{ms}-\mathrm{tz}}\left(\frac{516\xi_{\mathrm{ms}-\mathrm{tz}}^{2}+700t^{2}}{3\xi_{\mathrm{ms}-\mathrm{tz}}^{2}+175t^{2}}+\frac{129\xi_{\mathrm{ms}-\mathrm{ty}}^{2}+129\xi_{\mathrm{ms}-\mathrm{tz}}^{2}+175t^{2}}{3\xi_{\mathrm{ms}-\mathrm{ty}}^{2}+3\xi_{\mathrm{ms}-\mathrm{tz}}^{2}+175t^{2}}+\frac{129\xi_{\mathrm{ms}-\mathrm{tx}}^{2}+129\xi_{\mathrm{ms}-\mathrm{tz}}^{2}+175t^{2}}{3\xi_{\mathrm{ms}-\mathrm{tx}}^{2}+3\xi_{\mathrm{ms}-\mathrm{tz}}^{2}+175t^{2}}\right)$$

Based on Equations (14)–(18), the actuation forces, *f*_ac-tx_, *f*_ac-ty_ and *f*_ac-tz_, and the lost motions, δ_x_, δ_y_ and δ_z_, can be obtained when specific translational displacements of the MS, ξ_ms-tx_, ξ_ms-ty_ and ξ_ms-tz_, are required. Furthermore, the primary translational displacements of the ASs, ξ_asx-tx_, *ξ*_asy-tx_ and ξ_asz-tx_, can also be obtained according to Equation (4).

**Figure 11:**
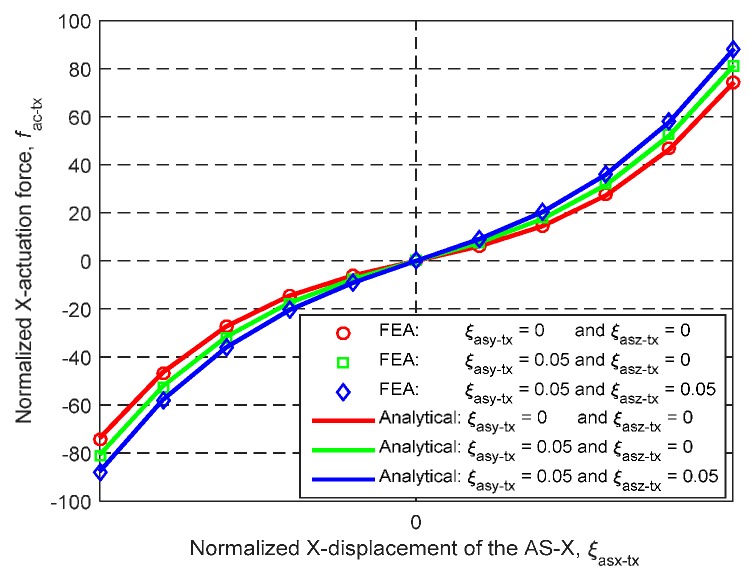
Analytical result and FEA result comparison regarding the relationship between the normalized X_ms_-axis primary translation of the AS-X and the normalized X_ms_-axis actuation force under the different conditions.

**Figure 12:**
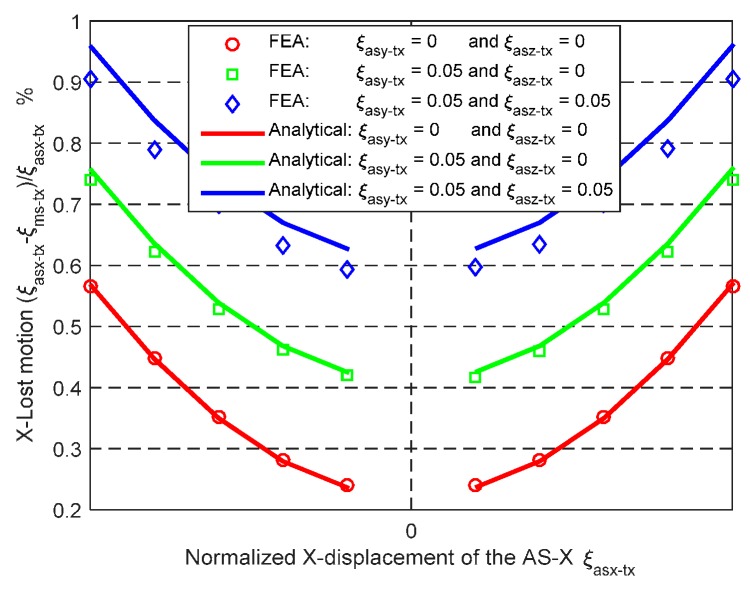
Analytical result and FEA result comparison regarding the relationship between the normalized X_ms_-axis primary translation of the AS-X and the X_ms_-axis lost motion under the different conditions.

The derived analytical models of the primary translations of the MS and ASs are applicable for any geometric dimension and material. When *L* = 30 mm, *W* = 20 mm, *T* = 1 mm, *E* = 69,000 MPa and *υ* = 0.33, the analytical and FEA results of the X_ms_-axis actuation force and the X_ms_-axis lost motion, can be seen in Figure 11 and Figure 12 under the following three actuation conditions: (a) ξ_asx-tx_ varies from −0.05 to +0.05, ξ_asy-tx_ = 0 and ξ_asz-tx_ = 0; (b) ξ_asx-tx_ varies from −0.05 to +0.05, ξ_asy-tx_ = 0.05 and ξ_asz-tx_ = 0; and (c) ξ_asx-tx_ varies from −0.05 to +0.05, ξ_asy-tx_ = 0.05 and ξ_asz-tx_ = 0.05. Figure 11 shows that the analytical results and the FEA results of the X_ms_-axis actuation force match very well, with less than 2% difference. Figure 12 illustrates that the maximum difference between the analytical results and the FEA results of the X_ms_-axis lost motion rate is less than 5.8%. It can be predicted that the analytical model of the MS’s primary translations should match the FEA model as well, according to Equation (4).

As studied in Section 4, the cross-axis coupling rate and the lost motion rate of the XYZ CPM along the X_ms_-axis are less than 0.38% and 0.68%, respectively, when λ_b_ = 60 and *w* = 1/3. Therefore, along one axis, the primary translations of the MS and the AS are almost decoupled from the primary translations of the MS and the ASs along the other two axes. However, the X_ms_-axis actuation forces, when ξ_asy-tx_ ≠ 0 and ξ_asz-tx_ ≠ 0, slightly deviate from the X_ms_-axis actuation force when ξ_asy-tx_ = 0 and ξ_asz-tx_ = 0, as shown in Figure 11. The deviation does not arise from the cross-axis coupling, but from the increase of the actuation stiffness along the X_ms_-axis due to the exerted actuation displacements, ξ_asy-tx_ and ξ_asz-tx_, along the Y_ms_- and Z_ms_-axes. The actuation stiffness along the three axes can be predicted by the derived analytical models, and the actuators can be selected based on the actuation stiffness.

## 6. Fabrication Considerations

The proposed new XYZ CPM has excellent motion performance. However, the MS of the XYZ CPM is located at the center of the whole structure. In order to fabricate the proposed XYZ CPM, two practical designs, design-I and design-II, of the proposed XYZ CPM are presented in this section.

**Figure 13:**
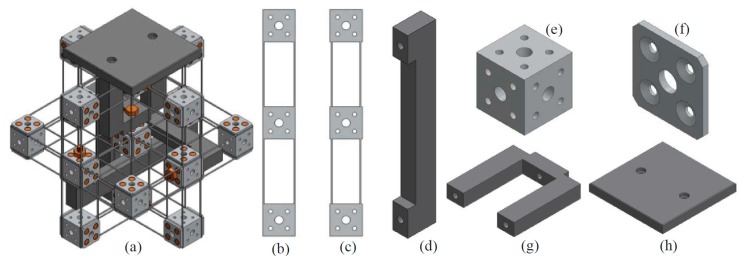
Practical design-I and its assembling accessories: (**a**) practical design-I, (**b**) compliant beam component with larger spanning size, (**c**) compliant beam component with smaller spanning size, (**d**) rigid cube linkage, (**e**) rigid cube, (**f**) washer, (**g**) output platform linkage and (**h**) output platform.

**Figure 14:**
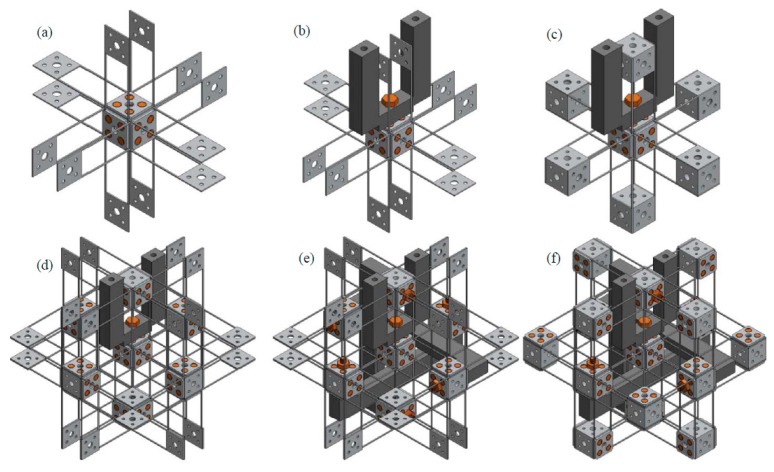
Assembling process of the design-I: (**a**) MS and its connected compliant beam components assembly, (**b**) output platform linkage assembly, (**c**) ASs assembly, (**d**) AS connected compliant beam components assembly, (**e**) rigid cube linkage assembly and (**f**) other rigid cubes assembly.

Design-I, as shown in Figure 13a, has an output platform, which is connected to the MS with an output platform linkage. Therefore, the motion of the MS can be transmitted to the output platform, *i.e.*, the output platform can translate in the three orthogonal directions under the actuation of the three actuators. Design-I is obtained by assembling the following basic accessories, as shown in Figure 13b–h, using bolts and nuts: compliant beam component with larger spanning size, compliant beam component with smaller spanning size, rigid cube linkage, rigid cube, washer, output platform linkage, and output platform. The two types of compliant beam components have different spanning size in order to prevent the interference between them in design-I. Both types of the compliant components are placed symmetrically in the practical design, so the small difference in the spanning size (the difference equals to twice the beam’s thickness) has a negligible effect on the motion characteristic. Each of the accessories has mounting holes which are used to assemble the accessory to design-I. The compliant beam components can be obtained through cutting a thin plate using a CNC milling machine, and the other basic accessories can also be fabricated using a CNC milling machine. The output platform is the last assembled accessory, and the other assembling steps are demonstrated in Figure 14.

Design-I has only seven different basic assembling accessories. Additionally, each of the basic assembling accessories can be fabricated easily. When one of the basic assembling accessories is broken, it is convenient to replace it with a new one. Moreover, different materials can be selected for the seven basic assembling accessories. For example, one can select Al6061 for the compliant beam components, select stainless steel for the rigid cubes and cube linkages, and select Polycarbonate for the output platform and its linkage.

**Figure 15:**
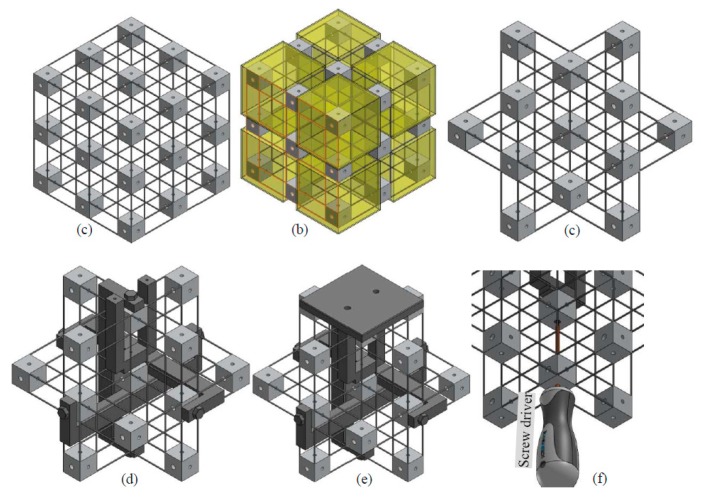
Manufacture and assembling process of the practical design-II: (**a**) monolithically fabricated model, (**b**) demonstration of the vertex cubes, (**c**) monolithically fabricated model without the vertex cubes, (**d**) output platform linkage and rigid cube linkage assembly, (**e**) output platform assembly and (**f**) demonstration of using a screwdriver to assemble the output platform linkage.

All of the rigid cubes and the compliant beam components can also be fabricated as a monolithic model, as shown in Figure 15a. The model can be fabricated through cutting along the three orthogonal directions, using electrical discharge machining technologies. If all the beams connecting to the eight vertex cubes of the model shown in Figure 15b are cut off, the model shown in Figure 15c can be obtained. Note that the associated rigid cubes should be fixed when cutting a compliant beam from them. The model, as shown in Figure 15c, is the key part of the practical design-II. Figure 15d–e illustrate the assembling of the output platform linkage, rigid cube linkages and output platform of design-II. Using a screwdriver to assemble the output platform linkage is also shown in Figure 15f. Compared with design-I, design-II has less assembling errors. However, the monolithic structure, as shown in Figure 15c, of the practical design-II has to be made of a single material, and the whole structure should be replaced if only one of the compliant beams is broken.

## 7. System Design

**Figure 16:**
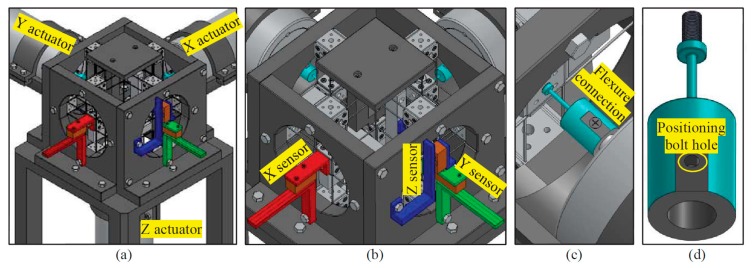
Demonstration of a spatial high-precision translational manipulator based on the practical design-I or design-II: (**a**) the spatial high-precision translational manipulator, (**b**) translational displacement measure sensor and (**c**,**d**) flexure actuator linkage.

The practical designs, design-I and design-II, have many potential applications such as micro-/nano-positioning platform and nano-manufacturing. This section presents a spatial high-precision translational manipulator, which is designed using the practical design-I or the practical design-II. The spatial high-precision translational manipulator is comprised of the practical design-I or the practical design-II, supporting stand, three actuators and displacement sensors, as shown in Figure 16a. The beam length, beam thickness and cube width of the spatial high-precision translational manipulator are 30, 1 and 20 mm, respectively. The peripheral dimension of the spatial high-precision translational manipulator is about 350 mm × 350 mm × 350 mm. This paper shows only the layout of the system design, while the detailed design for prototyping and experimental testing will be our further work. For the layout design, the following two key aspects should be emphasized: (a) displacement sensor assembling; and (b) actuator assembling.

In order to control the output platform at a specific position, the translational displacements of the output platform should be measured by a three-axis displacement sensor or three single-axis displacement sensors. If using three single-axis displacement sensors to measure the three translations of the output platform, each of the three single-axis displacement sensors should be capable of tolerating the other two displacements of the output platform. For convenience and decreasing the cost, we measure the displacements of the three rigid cube linkages instead of measuring the displacements of the output platform. The displacements of the three rigid cube linkages are almost the same as the displacements of the output platform along the three directions, respectively, because the lost motion rates of the proposed XYZ CPM are negligible. Furthermore, because each of the rigid cube linkages translates only along one specific direction, the displacement sensor measuring the displacement of the rigid cube linkage does not need to tolerate big transverse displacements along the other directions. Therefore, we can employ three accurate optical linear encoders to measure the displacements along the X-, Y- and Z-axes, respectively. Each optical linear encoder is comprised of a scale and a read head. The read head is used to read the encoded position from the scale. Figure 16b shows the assembly of the three optical linear encoders. The optical linear encoder, as shown in Figure 16b, is modeled based on a SIGNUM™ (Renishaw Public Limited Company, Gloucestershire, UK) RSLM/RELM high accuracy linear encoder system (the type number of the read head is A-9572-1514, and the type number of the scale is A-9660-0130), which can provide up to a 5-nm resolution.

Three cylindrical housed linear voice coil actuators (made by BEI Kimco Company located in Vista, CA, USA, and the type number of the actuator is LAH43-86-001Z) are selected to actuate the three translations of the high-precision spatial translational manipulator system, as shown in Figure 16a. The actuators can offer peak force 1512.4 N, continuous stall force 386.7 N and virtually unlimited resolution. The resolution is limited only by the associated displacement sensors. As analyzed in Section 4, the parasitic translations of the ASs are tiny. Therefore, the actuators do not suffer from off-axis displacements, even though the three actuators are rigidly connected to the three ASs, respectively. However, three flexure actuator linkages are still designed to connect the actuators with the ASs, which can be used to eliminate the assembly error effects of the actuators. Each of the flexure actuator linkages has a flexure beam, which is used to transmit the displacement from the actuator to the associated AS, as shown in Figure 16c. The flexure beam, as shown in Figure 16d, has one DOC along the beam and five DOF along and about other directions, so it can prevent the transmission of: (a) the assembly errors of the actuators to the ASs; and (b) the parasitic motions of the ASs to the actuators.

## 8. Conclusions

This paper proposed a new, compact and symmetric XYZ CPM, using the CPI approach with a configuration modification. The designed XYZ CPM not only provides translations along the three orthogonal directions, but also has desired motion characteristics such as reduced cross-axis coupling rates and minimized lost motion rates. It can be seen that the rigid stages of the XYZ CPM are over constrained by the wire beams, which is good for increasing the stiffness in the DOC directions. The stiffness in the DOF directions is also increased because of the overconstraints, which results in the increase of the actuation forces. However, the stiffness in the DOF directions is still small compared with the equivalent sheet-based XYZ CPMs. Moreover, the desired motion characteristics mentioned above were analyzed and validated by the FEA results, and the relations between the motion characteristics and the geometric parameters have been obtained. The primary translations of the XYZ CPM have also been analytically modeled, which can be used to quickly predict the actuation forces and actuation stiffness of an XYZ CPM. It can be seen that the good motion characteristics, especially the reduced cross-axis coupling rates and the minimized lost motion rates, can simplify the control system design.

Two practical designs of the new XYZ CPM were presented with consideration of the fabrication. The two practical designs can be used in many applications such as micro-/nano-manipulation. The layout design of a spatial high-precision translational system was also presented with consideration of the actuator and sensor integration. The further investigations including the physical prototyping, the experimental testing and the control system design, are our future work.
